# Twin pregnancy and early postpartum pelvic floor surface electromyographic activity: a single-center retrospective observational study using the glazer sEMG protocol

**DOI:** 10.3389/fmed.2026.1772761

**Published:** 2026-09-01

**Authors:** Shushu Yu, Xiaolei Chi, Xiang Ying, Xiaohan Su, Jiawen Yang, Yanlin Wang

**Affiliations:** 1 Department of Obstetrics and Gynecology, International Peace Maternity and Child Health Hospital, School of Medicine, Shanghai Jiao Tong University, Shanghai, China; 2 Shanghai Key Laboratory of Embryo Original Disease, Shanghai, China

**Keywords:** early postpartum period, Glazer protocol, pelvic floor surface electromyography, resting-phase sEMG parameters, twin pregnancy

## Abstract

**Background:**

Pregnancy and childbirth are established risk factors for postpartum pelvic floor dysfunction. However, the association between twin pregnancy and early postpartum pelvic floor surface electromyographic (sEMG) activity remains unclear. This study explored this association using the Glazer protocol.

**Methods:**

This retrospective study included 138 primiparous women with twin pregnancies and 540 with singleton pregnancies who underwent cesarean delivery. Pelvic floor sEMG was assessed at 42–60 days postpartum using a modified four-phase Glazer protocol. Between-group differences were examined using univariate analyses and multivariable linear regression. The primary model adjusted for maternal age, pre-pregnancy body mass index, gravidity, and mode of conception. Extended exploratory models additionally included pregnancy- and delivery-related variables and, subsequently, total neonatal birth weight as a potential mediator.

**Results:**

Compared with the singleton group, the twin group had lower pre- and post-resting mean sEMG amplitudes and higher pre- and post-resting phase scores (all *P* < 0.05). In the primary adjusted model, twin pregnancy was associated with lower pre-resting (β = −2.029 μV, 95% CI: −3.139 to −0.919; *P* < 0.001) and post-resting mean sEMG amplitudes (β = −1.310 μV, 95% CI: −2.327 to −0.293; *P* = 0.012), and higher pre-resting (β = 10.355, 95% CI: 3.967 to 16.742; *P* = 0.002) and post-resting phase scores (β = 9.011, 95% CI: 2.932 to 15.090; *P* = 0.004). In the extended model excluding total neonatal birth weight, associations with pre-resting amplitude and pre- and post-resting scores remained significant, whereas the post-resting amplitude association was no longer significant. After adjustment for total neonatal birth weight, none of the associations remained statistically significant.

**Conclusion:**

In general, the overall Glazer score in the early postpartum period was not significantly lower in women with twin pregnancies than in those with singleton pregnancies. In this exploratory study of primiparous women undergoing prelabor cesarean delivery, twin pregnancy was associated with differences in resting-phase sEMG parameters in the primary adjusted analysis. These associations were attenuated after adjustment for pregnancy-, delivery-, and birth weight–related variables, some of which may lie on the causal pathway. Prospective studies incorporating comprehensive pelvic floor assessments and clinically relevant outcomes are needed.

## 1 Introduction

Pelvic floor dysfunction (PFD) encompasses pelvic organ prolapse (POP), urinary incontinence (UI), fecal incontinence, and sexual dysfunction. Its prevalence varies according to population characteristics, including age and pregnancy or postpartum status. Previous studies have reported that approximately 50% of postpartum women experience symptoms related to PFD (1, 2). During pregnancy, maternal weight gain, fetal growth, and uterine enlargement increase intra-abdominal pressure and impose substantial mechanical loading on the pelvic floor muscles, nerves, and fascia. These structures may be compressed, stretched, or injured, thereby compromising pelvic floor support and function. During labor and vaginal delivery, prolonged second-stage labor and sustained pressure from the descending fetal head may further contribute to pelvic floor injury. Perineal tears, episiotomy, and assisted vaginal delivery may damage type I and type II pelvic floor muscle fibers and associated neural tissues, reduce pelvic floor muscle strength, and impair pelvic floor function (3, 4). In addition, advanced maternal age, overweight or obesity, chronic constipation, higher neonatal birth weight, parity, and multifetal pregnancy have been identified as potential risk factors for pelvic floor muscle injury and PFD (5). Therefore, pregnancy and childbirth are important contributors to postpartum pelvic floor dysfunction.

With the increasing use of assisted reproductive technology, the rate of twin pregnancies has risen over the past three decades. Compared with singleton pregnancies, twin pregnancies may impose greater mechanical stress on the pelvic floor because of greater total fetal weight and more pronounced uterine enlargement, potentially leading to more pronounced changes in pelvic floor support during pregnancy (6). Previous studies have suggested that women with twin pregnancies may be at increased risk of postpartum PFD, including POP and UI, compared with those with singleton pregnancies. In particular, the prevalence of UI during the first 20 weeks postpartum has been reported to be approximately twice as high after twin pregnancies as after singleton pregnancies, reaching 40% (7).

Although the effects of twin pregnancy on maternal health have been widely recognized, its association with pelvic floor function during the early postpartum period remains insufficiently understood. Previous studies have assessed pelvic floor outcomes using heterogeneous approaches, including self-reported symptoms, questionnaire-based measures, clinical examinations, and selected functional or imaging assessments. Although these approaches provide clinically relevant information, they evaluate different dimensions of pelvic floor health and may not directly reflect pelvic floor muscle electrical activity at rest or during voluntary contraction. Yang et al. reported that the Glazer assessment method was more reliable than manual muscle strength testing for evaluating pelvic floor function in postpartum women (8). Therefore, the present study used a modified four-phase Glazer protocol to assess pelvic floor surface electromyography (sEMG), aiming to provide a more objective evaluation of the association between twin pregnancy and early postpartum pelvic floor sEMG parameters.

## 2 Materials and methods

### 2.1 Study population

This single-center retrospective observational study included primiparous women who delivered at the International Peace Maternity and Child Health Hospital, Shanghai Jiao Tong University School of Medicine, between April 2021 and December 2023. Random sampling was not performed. Instead, all postpartum women who underwent pelvic floor function assessment at the Postpartum Pelvic Floor Rehabilitation Center 42–60 days after delivery and whose records could be linked to the obstetric medical record system were considered for inclusion.

At our institution, postpartum pelvic floor screening is provided as part of routine clinical care but is not a mandatory universal program. The service was suspended or restricted during part of 2022 because of COVID-19–related restrictions, resulting in fewer available assessment records during that period.

The same criteria were applied to both groups. Women were included if they were primiparous, aged 20–40 years, underwent cesarean delivery at ≥ 34 weeks of gestation without entering labor, underwent postpartum pelvic floor assessment 42–60 days after delivery, and had no history of pre-pregnancy pelvic floor dysfunction (PFD). Primiparity was defined as undergoing a first delivery; previous pregnancies ending in miscarriage or termination were not considered exclusion criteria. Exclusion criteria were age < 20 or > 40 years, gestational age < 34 weeks, pre-pregnancy PFD, multiparity, or labor before cesarean delivery. The two groups differed only in pregnancy type: women with twin pregnancies comprised the observation group, whereas women with singleton pregnancies comprised the control group.

After linkage of the pelvic floor screening database with the obstetric medical record system, 152 primiparous women with twin pregnancies and 589 primiparous women with singleton pregnancies were initially identified. After application of the eligibility criteria, 138 women with twin pregnancies and 540 women with singleton pregnancies were included in the final analysis.

### 2.2 Data collection

Data were obtained from two sources: the postpartum pelvic floor screening database and the obstetric electronic medical record database. The postpartum pelvic floor screening database included women who returned to the Postpartum Pelvic Floor Rehabilitation Center of our hospital for pelvic floor function assessment 42–60 days after cesarean delivery. This database contained basic demographic information, the Maternity Clinic Number, and Glazer assessment data. Surface electromyography of the pelvic floor muscles was performed by trained medical staff at the Pelvic Floor Screening and Rehabilitation Center using the Glazer protocol at 42–60 days postpartum.

The obstetric electronic medical record system included maternal age, gestational age, gravidity, mode of conception, education level, pre-pregnancy body mass, maternal body mass at delivery, gestational age at delivery, newborn birth weight, and pregnancy complications. In this study, the participants were primiparous women, defined as women undergoing their first delivery. However, gravidity could be greater than 1 because previous pregnancies ending in miscarriage or termination were also counted.

A total of 15,549 women who underwent cesarean delivery during the study period were initially identified from the obstetric electronic medical record system. After excluding multiparous women and those not meeting the eligibility criteria, the obstetric database contained 10,347 singleton primiparous women and 653 twin primiparous women. The pelvic floor screening database contained 1,341 records. The two databases were linked using the unique Maternity Clinic Number in Excel 2019 (Microsoft, Redmond, WA, USA). After linkage, 589 singleton primiparous women and 152 twin primiparous women were successfully matched and included in the final analysis. Unmatched records were excluded. Overall, 741 of 1,341 pelvic floor screening records (55.3%) were successfully matched to eligible obstetric records.

For quality control, all matched records were independently supplemented and verified against the original source data by two researchers. Records with duplicate or ambiguous identifiers were manually reviewed using the original obstetric and pelvic floor screening records, and any discrepancies were resolved by consensus.

### 2.3 Evaluation methods and evaluation indices

Pelvic floor sEMG was assessed using a neuromuscular stimulation instrument (SA9800, MLD B4; Medlander Medical Technology Inc., Nanjing, China) with a custom-made pear-shaped vaginal metal probe (CACB04, MLD V1; Medlander Medical Technology Inc., Nanjing, China). Signal analysis was performed using the MYOTRAC Infiniti system (Montreal, Canada), and all sEMG values were expressed in microvolts (μV). All assessments were conducted by professionally trained medical staff at the Pelvic Floor Screening and Rehabilitation Center of the International Peace Maternity and Child Health Hospital. Participants were examined in the supine position, and the pear-shaped vaginal metal probe was inserted into the vagina according to the standardized clinical procedure used at our center. Surface electrodes were placed over the hip adductor, gluteal, and abdominal muscles to monitor unwanted accessory muscle activation. sEMG measurements were conducted by two operators who had received professional training. Participants were randomly assigned to one of the two operators for the assessment. Prior to the examination, all participants were instructed to empty their bladder. Prior to formal testing, participants were informed of the complete examination procedure and received standardized instructions on correct pelvic floor muscle (PFM) contraction to minimize crosstalk from the abdominal and hip muscles. During the assessment, visual feedback and verbal cues were used to indicate the timing of contraction and relaxation. In addition, trained medical staff provided individualized pre-test guidance and practice to ensure that participants understood the procedure and were able to perform the required maneuvers correctly. The same examination procedure and standardized instructions were applied to all participants throughout the study to reduce operator- and participant-related variability. All participants successfully completed the assessment.

A modified four-phase protocol derived from the Glazer protocol (9) was used, with the sustained endurance contraction phase omitted. The protocol consisted of (1) a 60-s pre-baseline resting phase, during which participants were instructed to relax and remain aware of the PFM at rest. In accordance with commonly used clinical reference standards for pelvic floor sEMG, a mean resting EMG value of < 4 μV was considered to indicate normal resting muscle activity. (2) a fast-muscle assessment phase comprising five phasic (“flick”) contractions, with each contraction lasting 2 s and a 2-s rest interval between contractions. The maximum EMG value (μV) was defined as the highest peak amplitude among the five phasic contractions, whereas rise time (s) and recovery time (s) were calculated for each contraction and averaged across the five contractions; (3) a slow-muscle assessment phase comprising five tonic contractions, during which participants were instructed to contract the PFM as strongly as possible, hold each contraction for 10 s, and then fully relax for 10 s between contractions. The slow-muscle EMG value was defined as the mean EMG amplitude across the five tonic contractions; and (4) a 60-s post-baseline resting phase, during which participants were instructed to relax the PFM and resting EMG activity was recorded. Resting EMG values were averaged over the recording period. The mean EMG value over this period was also calculated, and a value of < 4 μV was considered within the normal resting range. Previous studies have also supported the reliability and reproducibility of pelvic floor sEMG assessment protocols (10). Unlike the standard five-phase Glazer protocol (9), the 60-s sustained endurance contraction phase was not included in this study because postpartum women may have reduced pelvic floor muscle endurance and may experience fatigue or discomfort during prolonged sustained contractions.

### 2.4 Covariates

Covariates were selected *a priori* based on the available literature. These included maternal characteristics (maternal age, pre-pregnancy BMI, education level, gestational weight gain, gravidity, and mode of conception), obstetric clinical conditions (hypertensive disorders of pregnancy, gestational diabetes mellitus, anemia of pregnancy, and thyroid disorders of pregnancy), gestational age at delivery, and birth weight. In this study, gravidity was defined as the total number of pregnancies, including previous pregnancies ending in miscarriage or termination; therefore, gravidity could be greater than 1 even among primiparous women.

Hypertensive disorders of pregnancy were defined according to the 2018 International Society for the Study of Hypertension in Pregnancy (ISSHP) criteria (11). Gestational diabetes mellitus was diagnosed according to the International Association of Diabetes and Pregnancy Study Groups (IADPSG) criteria based on a 75-g oral glucose tolerance test (12). Anemia of pregnancy was defined as a hemoglobin concentration of < 110 g/L (13). Thyroid disorders of pregnancy were diagnosed in accordance with the Guidelines for the Diagnosis and Management of Thyroid Disease during Pregnancy and Postpartum (2nd edition) (14).

### 2.5 Statistical analyses

Statistical analyses were performed using R software (version 4.3.3). Categorical variables were presented as frequencies and percentages. Continuous variables were expressed as mean ± standard deviation (SD) if normally distributed and as median (Q1, Q3) if non-normally distributed. Continuous variables were assessed for normality and compared using the independent-samples *t* test or Mann–Whitney U test, as appropriate. Categorical variables were compared using the chi-square test or Fisher’s exact test, as appropriate.

Multivariable linear regression analyses were performed to evaluate the association between twin pregnancy and postpartum pelvic floor sEMG parameters, with each Glazer protocol parameter treated as a continuous outcome. Singleton pregnancy was used as the reference group. Model 1 was adjusted for maternal age, pre-pregnancy body mass index (BMI), gravidity, and mode of conception. Model 2 was additionally adjusted for gestational age at delivery, gestational weight gain, anemia during pregnancy. Model 3 was further adjusted for total neonatal birth weight.

Regression coefficients (β), 95% confidence intervals (CIs), and two-sided P values are reported. Model assumptions were evaluated using standard regression diagnostic procedures, including assessments of linearity, normality of residuals, homoscedasticity, influential observations, and multicollinearity. All statistical tests were two-sided, and a *P*-value < 0.05 was considered statistically significant.

### 2.6 Reporting guideline

This retrospective cohort study was reported in accordance with the Strengthening the Reporting of Observational Studies in Epidemiology (STROBE) statement (15).

## 3 Results

**FIGURE 1:**
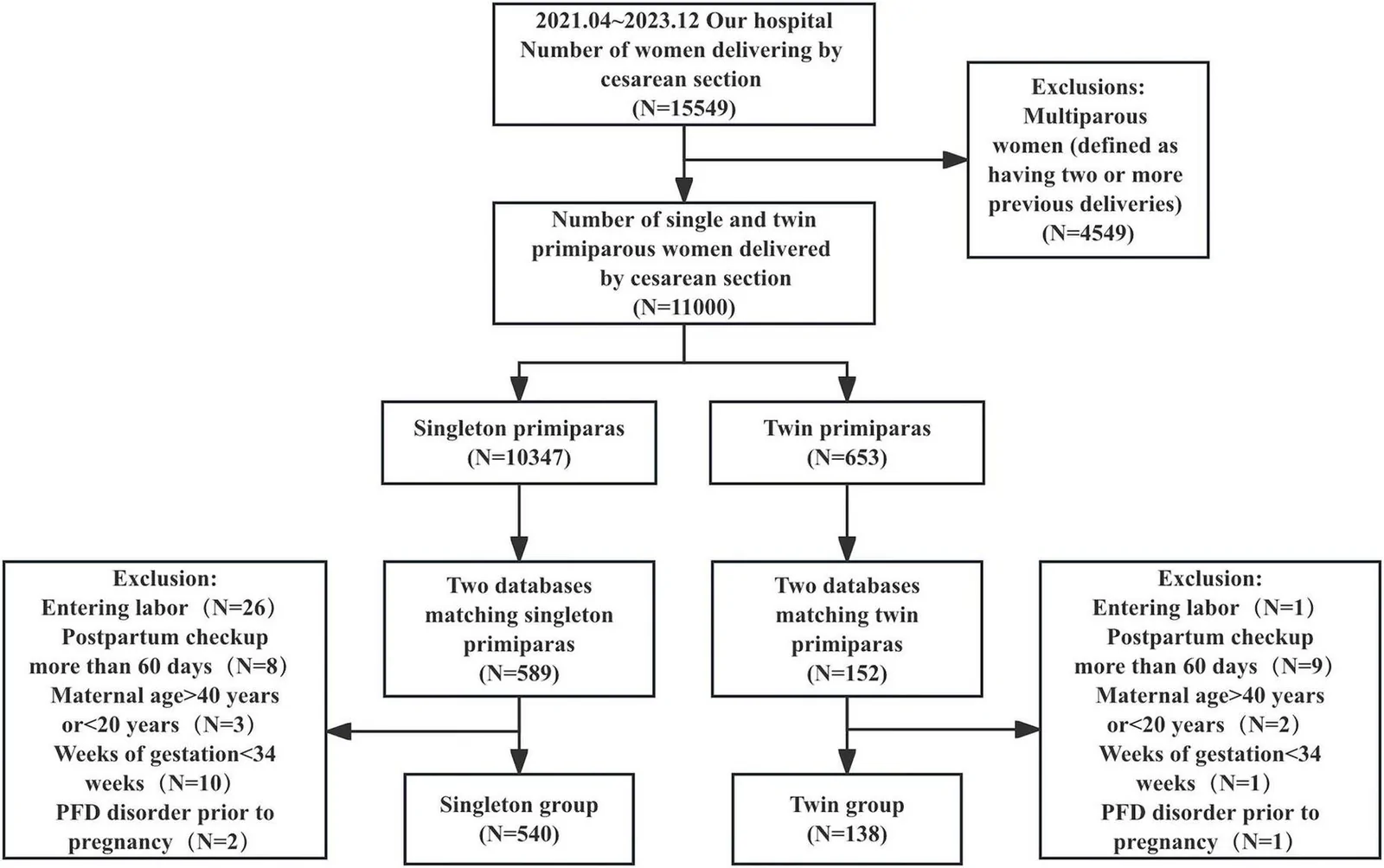
Flowchart of the subject screening process.

A total of 15,549 women who underwent cesarean delivery between April 2021 and December 2023 were initially identified. After application of the predefined eligibility criteria and linkage of the obstetric and pelvic floor screening databases, 678 primiparous women were included in the final analysis, including 540 women with singleton pregnancies and 138 women with twin pregnancies. The participant selection process and reasons for exclusion are shown in Figure 1.

The baseline characteristics of the study population are summarized in Table 1. Compared with the singleton group, women in the twin group were taller [163.8 (160.0–168.0) vs. 162.0 (159.0–166.0) cm, *P* = 0.040], had higher gravidity [1 (1–2) vs. 1 (1–1), *P* < 0.001], and were more likely to be aged ≥ 35 years (31.88% vs. 18.15%, *P* < 0.001). The proportion of women with gestational weight gain ≥ 20 kg was also higher in the twin group than in the singleton group (23.19% vs. 13.15%, *P* = 0.003). Women with twin pregnancies delivered at an earlier gestational age [36.6 (36.1–37.1) vs. 39.0 (38.2–39.6) weeks, *P* < 0.001] and had a higher total neonatal birth weight [4,990 (4,540–5,360) vs. 3,250 (2,965–3,580) g, *P* < 0.001] than those with singleton pregnancies. In addition, the prevalence of anemia during pregnancy was lower in the twin group than in the singleton group (19.57% vs. 30.00%, *P* = 0.015). Women in the twin group were more likely to have conceived using assisted reproductive technology (ART) than those in the singleton group (63.04% vs. 14.81%, *P* < 0.001). No statistically significant between-group differences were observed in pre-pregnancy BMI, BMI at delivery, educational level, hypertensive disorders of pregnancy, gestational diabetes mellitus, or thyroid disease during pregnancy (all *P* > 0.05).

**TABLE 1:** Demographic and clinical characteristics of the twin and singleton groups.

Characteristic	Singleton group (*n* = 540)	Twin group (*n* = 138)	*P*	SMD
Pre-pregnancy BMI (kg/m^2)^	21.43(19.53, 23.46)	20.76 (19.47, 22.51)	0.051	0.187
Gestational weight gain (kg)				
< 20	469 (86.85%)	106 (76.81%)	**0.003**	0.263
≥ 20	71 (13.15%)	32 (23.19%)		
BMI at delivery (kg/m^2^)	27.01 (24.53,29.46)	26.77 (25.63, 28.92)	0.931	0.089
Age (years)				
< 35	442 (81.85%)	94 (68.12%)	**<0.001**	0.321
≥ 35	98 (18.15%)	44 (31.88%)		
Height (cm)	162.0 (159.0,166.0)	163.8 (160.0, 168.0)	**0.040**	0.151
Educational level				
Junior college and less	102 (18.89%)	38 (27.54%)	0.067	0.206
Undergraduate	301 (55.74%)	72 (52.17%)		
Graduate and above	137 (25.37%)	28 (20.29%)		
Gravidity	1 (1,1)	1 (1,2)	**< 0.001**	0.736
Total birth weight (g)	3250 (2965,3580)	4990 (4540, 5360)	**< 0.001**	2.975
Gestational age at delivery (weeks)	39.0 (38.2,39.6)	36.6 (36.1, 37.1)	**< 0.001**	2.251
Hypertension in pregnancy				
No	436 (80.74%)	102 (73.91%)	0.099	0.164
Yes	104 (19.26%)	36 (26.09%)		
Anemia in pregnancy				
No	378 (70.00%)	111 (80.43%)	**0.015**	0.244
Yes	162 (30.00%)	27 (19.57%)		
Gestational diabetes				
No	433 (80.19%)	106 (76.81%)	0.381	0.082
Yes	107 (19.81%)	32 (23.19%)		
Thyroid disease in pregnancy				
No	446 (82.59%)	110 (79.71%)	0.508	0.070
Yes	94 (17.41%)	28 (20.29%)		
ART				
No	460 (85.19%)	51 (36.96%)	**< 0.001**	1.140
Yes	80 (14.81%)	87 (63.04%)		

BMI Body mass index; ART assisted reproductive technology. *P* < 0.05 are highlighted in bold text. Continuous variables are presented as median (IQR) and were compared between groups using the Mann-Whitney U test (Z-values reported). Categorical variables are presented as *n* (%) and were compared using the chi-square test (χ^2^ values reported). An absolute standardized mean difference (SMD) < 0.10 was considered indicative of negligible imbalance; an SMD of 0.10– < 0.20 indicated minor imbalance; and an SMD ≥ 0.20 indicated potentially meaningful imbalance.

As shown in Table 2, compared with the singleton group, the twin group exhibited lower mean sEMG amplitudes during both the pre-testing rest phase [5.09 (2.84–8.33) vs. 7.77 (4.46–11.68) μV, adjusted *P* < 0.001] and the post-testing rest phase [5.43 (3.52–9.13) vs. 7.38 (4.39–10.91) μV, adjusted *P* = 0.001]. In addition, the twin group had higher pre-resting phase scores [63 (30–85) vs. 36 (19–73), adjusted *P* < 0.001] and post-resting phase scores [59 (26–83) vs. 39 (19–72), adjusted *P* = 0.002] than the singleton group. No significant between-group differences were observed in the other Glazer assessment parameters, including fast- and slow-muscle stage sEMG parameters, overall score, fast-muscle stage score, and slow-muscle stage score, after adjustment for multiple comparisons (all adjusted *P* > 0.05).

**TABLE 2:** Comparison of Glazer assessment parameters between twin and singleton groups in the early postpartum period.

Stage/parameters	Singleton group (*n* = 540)	Twin group (*n* = 138)	*P*	Test statistic	Adjusted *P*
Pre-testing rest					
Average (μV)	7.77 (4.46, 11.68)	5.09 (2.84, 8.33)	**< 0.001**	47598	**< 0.001**
Variability	0.13 (0.11, 0.16)	0.14 (0.12, 0.16)	**0.047**	33192.5	0.124
Fast muscle (type II muscle fibers) stage					
Maximum (μV)	46.75 (35.38, 58.92)	43.51 (34.22, 55.00)	0.095	40684.5	0.191
Rise time (s)	0.35 (0.27, 0.46)	0.34 (0.28, 0.42)	0.325	39282.5	0.362
Recovery time (s)	0.42 (0.32, 0.56)	0.45 (0.32, 0.62)	0.268	34985	0.357
Slow muscle (type I muscle fibers) stage					
Average (μV)	32.16 (23.57, 40.39)	31.55 (22.28, 38.01)	0.138	40308.5	0.220
Variability	0.20 (0.17, 0.25)	0.21 (0.17, 0.27)	0.086	33742.5	0.191
Rise time (s)	0.39 (0.27, 0.60)	0.39 (0.25, 0.57)	0.309	39348	0.362
Recovery time (s)	0.79 (0.61, 1.06)	0.87 (0.65, 1.14)	**0.029**	32781	0.093
Post-testing rest					
Average (μV)	7.38 (4.39, 10.91)	5.43 (3.52, 9.13)	**< 0.001**	44999	**0.001**
Variability	0.13 (0.12, 0.17)	0.14 (0.12, 0.18)	0.153	34337.5	0.222
Overall score	71 (63, 77)	73 (62, 79)	0.34	35298	0.362
Pre-resting phase score	36 (19, 73)	63 (30, 85)	**< 0.001**	28020	**< 0.001**
Fast muscle (type II muscle fibers) stage score	89 (79, 94)	87 (79, 94)	0.505	38627.5	0.505
Slow muscle (type I muscle fibers) phase score	71 (60, 79)	71 (55, 78)	0.111	40535.5	0.197
Post-resting phase score	39 (19, 72)	59 (26, 83)	**0.001**	30160	**0.002**

*P* < 0.05 are highlighted in bold text. For the fast-muscle phase, maximum EMG (μV) represents the highest peak amplitude among the five phasic contractions, whereas rise time (s) and recovery time (s) represent the mean values across the five contractions. Adjusted *P* values were calculated using the Benjamini–Hochberg false discovery rate (FDR) method to correct for multiple comparisons across the Glazer parameters in the univariate analyses.

As shown in Table 3, singleton pregnancy was used as the reference group. In the primary minimally adjusted model (Model 1), adjusted for maternal age, pre-pregnancy BMI, gravidity, and mode of conception (ART), twin pregnancy was associated with a lower pre-resting phase mean sEMG amplitude (β = −2.029 μV, 95% CI: −3.139 to −0.919; *P* < 0.001) and a lower post-resting phase mean sEMG amplitude (β = −1.310 μV, 95% CI: −2.327 to −0.293; *P* = 0.012). Twin pregnancy was also associated with higher pre-resting phase scores (β = 10.355, 95% CI: 3.967 to 16.742; *P* = 0.002) and post-resting phase scores (β = 9.011, 95% CI: 2.932 to 15.090; *P* = 0.004). No significant associations were observed between twin pregnancy and pre-resting phase variability (β = 0.006, 95% CI: −0.026 to 0.039; *P* = 0.710) or slow-muscle phase recovery time (β = 0.124 s, 95% CI: −0.061 to 0.309; *P* = 0.187). In the secondary extended Model 2, which additionally adjusted for gestational age at delivery, gestational weight gain, anemia during pregnancy, and mode of delivery, twin pregnancy remained associated with a lower pre-resting phase mean sEMG amplitude (β = −1.730 μV, 95% CI: −3.098 to −0.363; *P* = 0.013), a higher pre-resting phase score (β = 10.868, 95% CI: 2.975 to 18.761; *P* = 0.007), and a higher post-resting phase score (β = 8.129, 95% CI: 0.617 to 15.641; *P* = 0.034). The association with post-resting phase mean sEMG amplitude was attenuated and was no longer statistically significant (β = −0.898 μV, 95% CI: −2.153 to 0.358; *P* = 0.161). In the further extended Model 3, which additionally included total neonatal birth weight, none of the associations between twin pregnancy and the examined pelvic floor Glazer parameters remained statistically significant (all *P* > 0.05).

**TABLE 3:** Associations between twin pregnancy and early postpartum pelvic floor Glazer parameters in sequential linear regression models.

Outcome	Model	β (95% CI)	*P-*value
Pre-resting phase mean (μV)	Crude	−2.381 (−3.303, −1.460)	**< 0.001**
	Model 1 (minimally adjusted)	−2.029 (−3.139, −0.919)	**< 0.001**
	Model 2 (extended, excluding total birth weight)	−1.730 (−3.098, −0.363)	0.013
	Model 3 (extended + total birth weight)	−2.057 (−4.422, 0.308)	0.088
Pre-resting phase variability	Crude	0.008 (−0.020, 0.035)	0.578
	Model 1 (minimally adjusted)	0.006 (−0.026, 0.039)	0.701
	Model 2 (extended, excluding total birth weight)	0.007 (−0.034, 0.047)	0.749
	Model 3 (extended + total birth weight)	−0.040 (−0.110, 0.030)	0.258
Slow muscle phase recovery time (s)	Crude	0.093 (−0.061, 0.248)	0.236
	Model 1 (minimally adjusted)	0.124 (−0.061, 0.309)	0.187
	Model 2 (extended, excluding total birth weight)	0.191 (−0.037, 0.420)	0.101
	Model 3 (extended + total birth weight)	0.137 (−0.258, 0.532)	0.496
Post-resting phase mean (μV)	Crude	−1.638 (−2.483, −0.792)	**< 0.001**
	Model 1 (minimally adjusted)	−1.310 (−2.327, −0.293)	**0.012**
	Model 2 (extended, excluding total birth weight)	−0.898 (−2.153, 0.358)	0.161
	Model 3 (extended + total birth weight)	−1.188 (−3.359, 0.983)	0.283
Pre-resting phase score	Crude	12.771 (7.464, 18.079)	**< 0.001**
	Model 1 (minimally adjusted)	10.355 (3.967, 16.742)	**0.002**
	Model 2 (extended, excluding total birth weight)	10.868 (2.975, 18.761)	**0.007**
	Model 3 (extended + total birth weight)	13.080 (−0.567, 26.728)	0.060
Post-resting phase score	Crude	10.078 (5.031, 15.125)	**< 0.001**
	Model 1 (minimally adjusted)	9.011 (2.932, 15.090)	**0.004**
	Model 2 (extended, excluding total birth weight)	8.129 (0.617, 15.641)	**0.034**
	Model 3 (extended + total birth weight)	8.298 (−4.693, 21.288)	0.210

Singleton pregnancy was used as the reference group. Regression coefficients (β) represent the mean difference in each outcome for women with twin pregnancy compared with women with singleton pregnancy. *P* < 0.05 are highlighted in bold text. Crude model: unadjusted. Model 1 (minimally adjusted model; primary model): adjusted for pre-exposure or baseline covariates selected *a priori* based on the directed acyclic graph, including maternal age, pre-pregnancy BMI, gravidity, and mode of conception (ART). Model 2 (extended model, excluding total birth weight): Model 1 additionally adjusted for gestational age at delivery, gestational weight gain, anemia in pregnancy. Model 3 (extended model plus total birth weight): Model 2 additionally adjusted for total neonatal birth weight. Models 2 and 3 are secondary exploratory analyses, as pregnancy-related variables may lie on the causal pathway between twin pregnancy and postpartum pelvic floor outcomes. CI, confidence interval; BMI, body mass index; ART, assisted reproductive technology.

## 4 Discussion

The pelvic floor muscles are often described as a “muscular hammock” that contributes to the support of the uterus, bladder, rectum, and other pelvic organs. They also participate in continence, defecation, and sexual function. Postpartum pelvic floor disorders, including urinary incontinence and pelvic organ prolapse, are common and may substantially affect women’s quality of life. In recent decades, the rate of twin births has increased globally, partly because of delayed childbearing and the widespread use of assisted reproductive technology (ART). Changes in pelvic organ support during the early postpartum period may be related not only to labor and delivery but also to physiological and mechanical changes occurring during pregnancy (16). Compared with singleton pregnancy, twin pregnancy is associated with greater uterine enlargement, increased intra-abdominal pressure, greater maternal weight gain, and potentially greater mechanical loading of the pelvic floor and pelvic support structures (6). However, evidence regarding the association between twin pregnancy and early postpartum pelvic floor muscle sEMG activity remains limited.

In this single-center retrospective study, twin pregnancy was associated with distinct resting-phase sEMG patterns compared with singleton pregnancy after adjustment for pre-exposure confounders, characterized by lower mean amplitudes and higher corresponding phase scores. Notably, these associations were progressively attenuated after adjustment for pregnancy- and delivery-related variables and were no longer present after further adjustment for total neonatal birth weight. This stepwise attenuation suggests that pregnancy-related factors—particularly cumulative fetal load—may lie on the causal pathway linking twin pregnancy to postpartum resting sEMG activity. Furthermore, the absence of between-group differences in fast- and slow-muscle phase scores indicates that the observed electrophysiological differences were specific to the resting state rather than reflecting generalized differences in pelvic floor muscle performance, a distinction that may inform the interpretation of sEMG findings in postpartum assessment.

Several factors may contribute to the observed resting-phase differences. Women with twin pregnancies delivered at earlier gestational ages, which may have resulted in a shorter duration of mechanical stress on the pelvic floor structures. In addition, hormonal differences may play a role: maternal serum estradiol levels increase with fetal number, and estradiol concentrations are higher in multiple pregnancies (17, 18). The substantially higher rate of ART conception in the twin group, with ovarian stimulation associated with elevated estradiol levels (19, 20), further supports this possibility. Estrogen receptors are widely distributed in pelvic floor tissues (21), and experimental evidence suggests that estrogen influences connective tissue metabolism and neuromuscular activity (22–26). However, hormonal levels were not measured in this study, and the relative contributions of mechanical versus hormonal factors to the observed sEMG differences warrant further investigation.

Previous studies have reported higher rates of urinary incontinence and pelvic organ prolapse after twin pregnancies (7, 27–29). These studies mainly evaluated clinical symptoms, whereas the present study assessed sEMG parameters. sEMG is a noninvasive technique that records electrical activity through intravaginal or anal electrodes (10) and provides complementary information on pelvic floor muscle activation in postpartum assessment and rehabilitation settings (9, 30). Previous research has reported a high prevalence of postpartum electrophysiological abnormalities, although definitions and protocols vary across studies (31). The observed resting-phase differences do not directly establish differences in pelvic floor contractile capacity, support function, or future risk of pelvic floor disorders. However, they provide electrophysiological evidence that twin pregnancy is associated with distinct resting neuromuscular activity patterns, extending the existing literature that has primarily focused on clinical symptoms.

Clinically, the present findings suggest that women with twin pregnancies may exhibit distinct resting sEMG patterns in the early postpartum period. However, sEMG should be interpreted alongside clinical symptoms, digital examination, and ultrasound, as apparently acceptable electrophysiological findings may coexist with underlying connective tissue changes. The specificity of the findings to the resting phase suggests that resting sEMG parameters and voluntary contractile parameters may capture different dimensions of pelvic floor neuromuscular function, which may have implications for postpartum rehabilitation planning.

Several limitations should be considered. First, the retrospective single-center design may limit generalizability. Second, the exact assessment day within the 42–60 day window was not systematically recorded, potentially introducing variability. Third, the commercial vaginal probe (CACB04, MLD V1; Medlander Medical Technology Inc., Nanjing, China) used for sEMG acquisition has not been directly validated against the original Glazer sensor in published head-to-head studies; thus, absolute μV values should be interpreted within the context of this study rather than compared with values from other devices. Fourth, sEMG reflects neuromuscular activation rather than structural integrity, and occult connective tissue changes may remain undetected. Fifth, the absence of hormonal measurements and long-term follow-up precludes assessment of hormonal influences and long-term outcomes.

In this single-center retrospective study, twin pregnancy was associated with distinct resting-phase sEMG patterns compared with singleton pregnancy after adjustment for pre-exposure confounders, characterized by lower mean amplitudes and higher corresponding phase scores. Notably, these associations were progressively attenuated after adjustment for pregnancy- and delivery-related variables and were no longer present after further adjustment for total neonatal birth weight. This stepwise attenuation suggests that pregnancy-related factors—particularly cumulative fetal load—may lie on the causal pathway linking twin pregnancy to postpartum resting sEMG activity. Furthermore, the absence of between-group differences in fast- and slow-muscle phase scores indicates that the observed electrophysiological differences were specific to the resting state rather than reflecting generalized differences in pelvic floor muscle performance, a distinction that may inform the interpretation of sEMG findings in postpartum assessment.

Several factors may contribute to the observed resting-phase differences. Women with twin pregnancies delivered at earlier gestational ages, which may have resulted in a shorter duration of mechanical stress on the pelvic floor structures. In addition, hormonal differences may play a role: maternal serum estradiol levels increase with fetal number, and estradiol concentrations are higher in multiple pregnancies (17, 18). The substantially higher rate of ART conception in the twin group, with ovarian stimulation associated with elevated estradiol levels (19, 20), further supports this possibility. Estrogen receptors are widely distributed in pelvic floor tissues (21), and experimental evidence suggests that estrogen influences connective tissue metabolism and neuromuscular activity (22–26). However, hormonal levels were not measured in this study, and the relative contributions of mechanical versus hormonal factors to the observed sEMG differences warrant further investigation.

Previous studies have reported higher rates of urinary incontinence and pelvic organ prolapse after twin pregnancies (7, 27–29). These studies mainly evaluated clinical symptoms, whereas the present study assessed sEMG parameters. sEMG is a noninvasive technique that records electrical activity through intravaginal or anal electrodes (10) and provides complementary information on pelvic floor muscle activation in postpartum assessment and rehabilitation settings (9, 30). Previous research has reported a high prevalence of postpartum electrophysiological abnormalities, although definitions and protocols vary across studies (31). The observed resting-phase differences do not directly establish differences in pelvic floor contractile capacity, support function, or future risk of pelvic floor disorders. However, they provide electrophysiological evidence that twin pregnancy is associated with distinct resting neuromuscular activity patterns, extending the existing literature that has primarily focused on clinical symptoms.

Clinically, the present findings suggest that women with twin pregnancies may exhibit distinct resting sEMG patterns in the early postpartum period. However, sEMG should be interpreted alongside clinical symptoms, digital examination, and ultrasound, as apparently acceptable electrophysiological findings may coexist with underlying connective tissue changes. The specificity of the findings to the resting phase suggests that resting sEMG parameters and voluntary contractile parameters may capture different dimensions of pelvic floor neuromuscular function, which may have implications for postpartum rehabilitation planning.

Several limitations should be considered. First, the retrospective single-center design may limit generalizability. Second, the exact assessment day within the 42–60 day window was not systematically recorded, potentially introducing variability. Third, the commercial vaginal probe (CACB04, MLD V1; Medlander Medical Technology Inc., Nanjing, China) used for sEMG acquisition has not been directly validated against the original Glazer sensor in published head-to-head studies; thus, absolute μV values should be interpreted within the context of this study rather than compared with values from other devices. Fourth, sEMG reflects neuromuscular activation rather than structural integrity, and occult connective tissue changes may remain undetected. Fifth, the absence of hormonal measurements and long-term follow-up precludes assessment of hormonal influences and long-term outcomes.

In summary, twin pregnancy was associated with differences in resting-phase sEMG parameters after adjustment for pre-exposure confounders, but these associations were attenuated after adjustment for pregnancy-related variables and were no longer significant after adjustment for total neonatal birth weight, suggesting that cumulative fetal load may contribute to the observed electrophysiological differences. Prospective longitudinal studies with standardized assessments, hormonal measurements, and long-term clinical follow-up are needed to validate these findings and establish their clinical relevance.

## 5 Conclusion

In general, the overall Glazer score did not differ significantly between twin and singleton pregnancies. However, in this exploratory study of primiparous women undergoing prelabor cesarean delivery, twin pregnancy was associated with differences in resting-phase sEMG parameters in the primary adjusted analysis. These associations were attenuated after adjustment for pregnancy-, delivery-, and birth weight–related variables, some of which may lie on the causal pathway. As sEMG measures muscle electrical activity rather than comprehensive pelvic floor function, validated minimal clinically important differences for postpartum Glazer parameters are unavailable. Prospective studies incorporating comprehensive pelvic floor assessments and clinically relevant outcomes are needed.

## Data Availability

The raw data supporting the conclusions of this article are not publicly available due to ethical/privacy restrictions. However, de-identified data can be made available by the corresponding author upon reasonable request.
